# Case of Scirrhus of the Liver in a Child Three Months Old

**Published:** 1857-05

**Authors:** Samuel W. Gross

**Affiliations:** Philadelphia


ART. V.—*Case of Scirrhus of the Liver in a Child three months old.* By SAMUEL W. GROSS, M.D., of Philadelphia.

THE subject, a colored boy, aged three months, for three weeks before death had suffered from vomiting, cough, and shortness of breath to such an extent, that its mother supposed the immediate cause of its decease was suffocation. Its appetite was unimpaired and the abdomen was in a swollen condition.

An autopsy was made eight hours after death by my father and myself. The peritoneum contained a small quantity of fluid, and evidences of recent inflammation existed in the lesser omentum; but none upon the parietal portion of the membrane immediately in contact with the scirrhous masses. The mesenteric ganglia were somewhat enlarged; but with the exception of the liver, the abdominal, as well as the thoracic viscera, were in a normal condition. A little serum was found in the pericardium.

I may remark that the child had double congenital scrotal hernia along with hydrocele, and that the inguinal rings were very patulous.

The liver was of the natural volume, of good color, and of a firm consistence. On the anterior margin of the left lobe were three scirrhous masses, each about one inch and three quarters in length, half an inch in thickness, and from half an inch to an inch and a half in width, being separated from each other by fissures in the hepatic structure. They were flattened, but projected somewhat above the surface of the liver; and had very much the appearance and consistence of fibro-cartilage, grating slightly under the knife, and being separated from the surrounding healthy tissue by a distinct irregular lunated line of demarcation, which was of a dark brownish-red color. Six other patches, varying in size from a shot to that of a three-cent piece, were situated upon the upper surface of this lobe, and were interspersed with minute white spots. Upon its under surface, at the middle of the longitudinal fissure, was a single conical mass, about the size of a small hazel-nut.

The anterior border of the right lobe was to some extent pervaded by small points, while its body was occupied by three tumors, of a spherical shape, and varying in size from a hemp-seed to that of a hazel-nut.

The gall-bladder contained about half a drachm of thin, greenish bile. Examined with the microscope, the scirrhous structure was found to be remarkably distinct; but no one who is familiar with this kind of deposit could have mistaken it by mere inspection, so well marked were its external characters.

